# Alteration of Basal Ganglia and Right Frontoparietal Network in Early Drug-Naïve Parkinson’s Disease during Heat Pain Stimuli and Resting State

**DOI:** 10.3389/fnhum.2015.00467

**Published:** 2015-08-24

**Authors:** Ying Tan, Juan Tan, Jiayan Deng, Wenjuan Cui, Hui He, Fei Yang, Hongjie Deng, Ruhui Xiao, Zhengkuan Huang, Xingxing Zhang, Rui Tan, Xiaotao Shen, Tao Liu, Xiaoming Wang, Dezhong Yao, Cheng Luo

**Affiliations:** ^1^Key Laboratory for NeuroInformation of Ministry of Education, High-Field Magnetic Resonance Brain Imaging Key Laboratory of Sichuan Province, Center for Information in Medicine, University of Electronic Science and Technology of China, Chengdu, China; ^2^School of Computer Science and Technology, Southwest University for Nationalities, Chengdu, China; ^3^Neurology Department, Affiliated Hospital of North Sichuan Medical College, North Sichuan Medical College, Nanchong, China; ^4^School of Life and Science Engineering, Southwest Jiaotong University, Chengdu, China

**Keywords:** Parkinson’s disease, fMRI, functional connectivity, functional network connectivity, basal ganglia network, right frontoparietal network

## Abstract

**Background:**

The symptoms and pathogenesis of Parkinson’s disease (PD) are complicated and an accurate diagnosis of PD is difficult, particularly in early-stage. Because functional magnetic resonance imaging (fMRI) is non-invasive and is characterized by the integration of different brain areas in terms of functional connectivity (FC), fMRI has been widely used in PD research. Non-motor symptom (NMS) features are also frequently present in PD before the onset of classical motor symptoms with pain as the primary NMS. Considering that PD could affect the pain process at multiple levels, we hypothesized that pain is one of the earliest symptoms in PD and investigated whether FC of the pain network was disrupted in PD without pain. To better understand the pathogenesis of pain in PD, we combined resting state and pain-stimuli-induced task state fMRI to identify alterations in FC related to pain in PD.

**Methods:**

Fourteen early drug-naïve PD without pain and 17 age- and sex-matched healthy controls (HC) participated in our testing task. We used independent component analysis to select seven functional networks related to PD and pain. We focused on abnormalities in FC and in functional network connectivity (FNC) in PD compared with HC during the task (51°C heat pain stimuli) and at rest.

**Results:**

Compared with HC, PD showed decreased FC in putamen within basal ganglia network (BGN) in task state and decreased FC in putamen of salience network (SN) and mid-cingulate cortex of sensorimotor network in rest state. FNC between the BGN and the SN are reduced during both states in PD compared with HC. In addition, right frontoparietal network (RFPN), which is considered as a bridge between the SN and default-mode network, was significantly disturbed during the task.

**Conclusion:**

These findings suggest that BGN plays a role in the pathological mechanisms of pain underlying PD, and RFPN likely contributes greatly to harmonization between intrinsic brain activity and external stimuli.

## Introduction

Parkinson’s disease (PD) is an age-related neurodegenerative disorder that is the second most prevalent neurodegenerative disorder, after Alzheimer’s disease. PD is a multi-system disorder that is characterized by a combination of motor and non-motor symptoms (NMSs) (Khoo et al., 2013). The hallmarks of motor symptoms are resting tremors, rigidity, postural disabilities, and NMS, including cognitive impairment, sleep disorders, dysautonomia, and pain.

Making an accurate diagnosis of PD, particularly in its early stages, is difficult. An urgent need exists to develop early diagnostic markers to intervene at the onset of disease and slow or stop the course of the disease (Miller and O’Callaghan, 2015). PD has been studied from clinical, imaging, pathological, biochemical, and genetic perspectives (Brooks and Pavese, 2011; Schapira, 2013; Sharma et al., 2013; Lin and Wu, 2015; Miller and O’Callaghan, 2015). Most studies proved that PD is characterized by early prominent loss of dopaminergic neurons. Currently, structural and functional neuroimagings, including positron emission tomography (PET), single photon emission computer tomography (SPECT), and functional magnetic resonance imaging (fMRI), are available to detect pathophysiological and biochemical changes associated with PD and to follow disease progression (Brooks and Pavese, 2011; Miller and O’Callaghan, 2015). SPECT and PET are methods for assessing the density of presynaptic dopaminergic terminals within the striatum (Thobois et al., 2001); however, both methods involve the use of radioactive nuclides from either natural or synthetic sources. Nevertheless, fMRI retains a role for non-invasive, high spatial resolution. With the rapid evolution of advanced magnetic resonance imaging (MRI) techniques in the last decades, fMRI has allowed us to further investigate PD, the progressive degenerative neurological disorder (Husarova et al., 2014; Tessitore et al., 2014).

Furthermore, fMRI can characterize the integration of different brain areas in terms of functional connectivity (FC) (Friston, 2009; Luo et al., 2011a). Studies of resting fMRI have reported alterations of FC in the default-mode network (DMN) and the subcortical and/or cortical motor network in PD (Hacker et al., 2012; Yoo et al., 2015). The increased strength of cortico-striatal FC in PD was observed, particularly within motor cortical regions (Kwak et al., 2010). The presence of mild cognitive impairment at early stages of PD affects activity in the prefrontal cortex and caudate nucleus as well as motor-related regions (Nagano-Saito et al., 2014). Luo et al. (2015) demonstrated that a disruption of whole-brain topological organization might contribute to preclinical changes in early stage drug-naive PD patients by investigating resting state functional brain networks.

Non-motor symptom features are frequently present in PD before the onset of the classical motor symptoms (Pont-Sunyer et al., 2015). Recent epidemiological studies have shown that 70–80% of patients with PD suffer from painful sensations. PD could affect the pain process at multiple levels, from transmission of pain from peripheral structures to the higher centers, reception and interpretation of pain, as well as interference with several anatomic structures involved in pain mechanisms. Pain is one of the earliest symptoms in PD (Fil et al., 2013). We hypothesized that the FC of pain networks was disrupted in PD patients without pain. Therefore, we investigated the FC differences during a resting state and a heat-induced pain-stimuli task state fMRI in the brain areas of two groups: a control group and a group of early drug-naïve PD patients without pain.

Functional connectivity is defined as a correlation among spatially remote brain regions (Friston, 2002; Arbabshirani et al., 2013). The independent component analysis (ICA) method, using blind source separation techniques, deconstructs the entire BOLD data set into statistically independent components (ICs) (Hyvarinen and Oja, 2000; Luo et al., 2014a) and models functional brain networks without an *a priori* functional hypothesis. FC has been used to research functional connections within brain networks. Functional network connectivity (FNC) (Jafri et al., 2008; Luo et al., 2011b) measures FC among brain networks. Recently, investigations of FC and FNC have focused on comparing either two participant groups or two experimental states (Sporns, 2014; Li et al., 2015). Fewer studies investigated the FC and FNC of brain networks by comparing two participant groups (PD and control groups) and two experimental states (resting and pain-stimuli states) simultaneously in PD research.

## Materials and Methods

### Subjects

Thirty-one subjects (14 patients with idiopathic early stage PD without pain and 17 healthy subjects) participated in this investigation. All were right handed. The study was approved by the Ethics Committee of the North Sichuan Medical College Affiliated Hospital, and written informed consent was obtained from all subjects.

Subjects in the early PD group were diagnosed by movement disorder neurologists and met the UK PD Society Brain Bank diagnostic criteria (Hughes et al., 1992). All patients were assessed using the Unified Parkinson’s Disease Rating Scale (UPDRS) and the Mini-Mental State Examination (MMSE) and had not been treated with anti-Parkinson medications prior to the initial visit (i.e., drug naive). Patients with a history of head injury, moderate–severe head tremor, stroke, and other neurological diseases or disorders that interfered with assessing manifestations of PD were excluded from the study.

The control group comprised age- and sex-matched healthy participants with no historical or current diagnosis according to the Diagnostic and Statistical Manual of Mental Diseases Axis-V, no neurological illnesses and no brain lesions. To exclude any relevant structural abnormalities, all subjects underwent MRI before joining the study.

### Task design

Two sets of fMRI data were collected from all subjects from one task session of heat pain stimuli (51°C) and one resting state session. Thermal sensation and pain were measured using a contact heat evoked potential stimulator (CHEPS; Medoc Ltd., Ramat Yishai, Israel). One end of the CHEPS probe contains a thermode (area = 572.5 mm^2^) and a heating thermo-foil (Minco Products, Inc., Minneapolis, MN, USA) covered with a 25-μm layer of thermal conductive plastic. The CHEPS thermode was placed in direct contact with the right dorsal forearm. Sophisticated pain stimulators were integrated with the fMRI system and synchronized with its main computer. All stimuli were initiated from a baseline temperature of 32°C to a target temperature of 51°C applied by computer-controlled brief radiant pulses with rapid change (rise rate: 70°C/s). Twenty stimuli were included in the task state. The interval between stimuli was randomly varied between 16, 20, and 24 s to avoid habituation. The participants also underwent a 410-s scan during the resting state. During data acquisition, the participants were asked to relax with their eyes closed without falling asleep.

### Image acquisition

All MRI data sets were scanned in a 3-T MRI scanner (EXCITE, HDx, Software 14X.M5, General Electric Healthcare, Milwaukee, WI, USA) with a 32-channel phased array head coil in Affiliated Hospital of North Sichuan Medical College. Anatomical T1-weighted images were acquired using a three-dimensional (3D)-spoiled gradient recalled (SPGR) sequence, generating 156 axial slices [thickness: 1 mm (no gap), TE = 3.2 ms, TR = 8.2 ms, FOV = 240 mm × 240 mm, flip angle = 12°, matrix = 256 × 256]. The fMRI data were acquired using gradient echo-planar imaging (EPI) sequences. The imaging parameters were as follows: thickness = 4 mm (no gap); TE = 30 ms; TR = 2,000 ms; FOV = 240 mm × 240 mm; flip angle = 90°; and matrix = 64 × 64. Two hundred five volumes (35 slices per volume) were obtained during the 410 s of an fMRI session. To ensure steady-state longitudinal magnetization, the first 5 volumes were excluded.

### Data analysis: Preprocessing

All fMRI data were preprocessed using the SPM8 software package (statistical parametric mapping)^1^. All data were slice timing and motion corrected, spatially normalized into standard MNI space, and the image was resampled to 3 mm × 3 mm × 3 mm. Head motion was restricted to <1.5 mm of displacement or 1 degree of rotation in any direction. No subject was excluded. Finally, spatial smoothing was performed with an 8-mm full-width half-maximum Gaussian kernel.

### Connectivity analysis

All processed data from the resting state and the task state of the PD and control groups were analyzed using a group ICA from the fMRI toolbox (GIFT version 1.3)^2^. IC estimation included three steps: data reduction, application of the ICA algorithm and back-reconstruction (Calhoun et al., 2001). The data dimensionality was reduced using two steps of principal component analysis, and the optimal number of IC was estimated using the minimum description length (MDL) algorithm (Li et al., 2007). Next, the Informix algorithm (Bell and Sejnowski, 1995) was used to run the ICA. To determine the robustness of the ICA, the analysis was repeated 30 times using ICASSO. Finally, the temporospatial back-reconstruction method was used to generate time courses and spatial maps for each participant.

### Functional network connectivity

Although many of ICs represents the brain networks with spatial independent each other, temporal correlations may exist among the time courses. We used the FNC toolbox^3^ to compute the maximum lagged correlation among the selected intrinsic connectivity networks (ICNs).

The maximum correlation and corresponding lag were calculated for each of the subjects and for both states in the FNC analysis. According to previous studies (Luo et al., 2012; Li et al., 2015), the lag was set in an arrangement within −5 to 5 s.

### Statistical analysis

To determine the effects of group and state, we performed a two-way repeated-measures analysis of variance (ANOVA) with group [PD and healthy control (HC)] as a between-subject factor and state (task and rest) as a repeated-measures factor. Statistical analyses were performed at the voxel-wise level and at the network level.

The significance threshold of group differences of FC maps (voxel-wise level) was set to *p* < 0.01 (Alphasim corrected). The significance threshold of group differences of FNC (the network level) was set to *p* < 0.05 [false discovery rates (FDR) correction].

For those clusters and networks showing significant main effects and interaction between group and state, *post hoc* paired *t* tests and two-sample *t* tests were performed.

## Results

### Demographics and ICNs

The demographics and clinical characteristics of subjects are shown in Table 1. Forty-seven ICs were decomposed using Spatial ICA in GIFT for rest and task. All of these components were stable across multiple runs of IC decomposition (stability index assessed by ICASSO ranging from 0.95 to 0.98).

**Table 1:** **Demographics, clinical characteristics**.

	PD (*n* = 14)	HC (*n* = 17)	
		
	Means ± SD	Means ± SD	*p* value
Age (years)	62.79 ± 4.59	61.35 ± 4.27	0.376
Gender^a^	11 M/3 F	13 M/4 F	0.889
Disease duration (years)	2.46 ± 1.43	–	–
UPDRS score			
Part I – mood/cognition	2.93 ± 1.14	–	–
Part II – activities of daily living	9.93 ± 2.27	–	–
Part III – motor examination	21.79 ± 5.66	–	–
Part IV – complications of therapy	0	–	–
MMSE score	23.93 ± 2.13	–	–

*^a^Chi-square test*.

*PD, Parkinson’s disease; HC, healthy controls; M, male; F, female; UPDRS, Unified Parkinson’s Disease Rating Scale; MMSE, Mini-Mental State Exam*.

**Figure 1:**
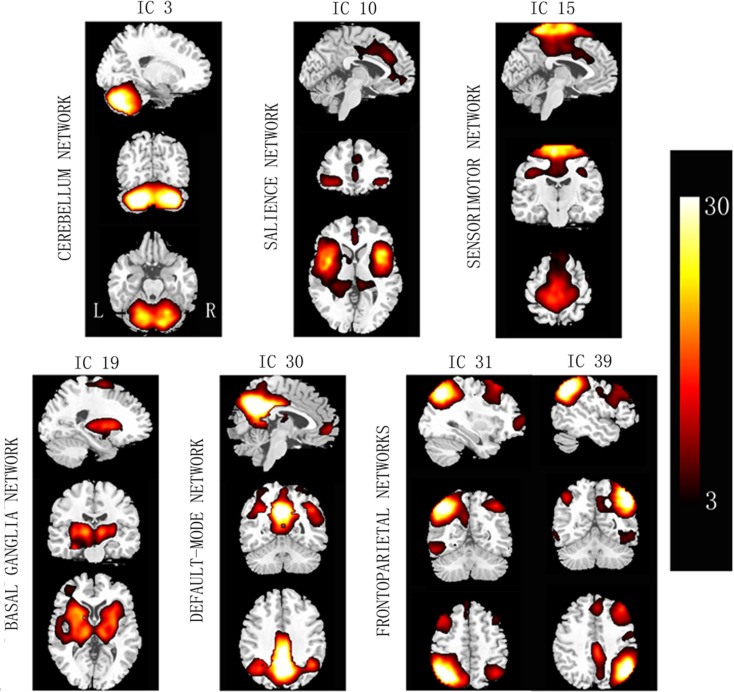
**Spatial maps of the seven selected independent components**. Column bar shows *t* contrast threshold. L, left; R, right.

Based on the experience of pain-related and PD-related ICNs (Wu et al., 2009; Otti et al., 2013; Szewczyk-Krolikowski et al., 2014; Baggio et al., 2015), 7 of the 47 ICA components were inspected visually for confirmation of non-artifact changes. These components included the basal ganglia network (BGN, IC #19), the right frontoparietal network (RFPN, IC #31), the salience network (SN, IC #10), the DMN (IC #30), the sensorimotor network (SMN, IC #15), the cerebellum network (CN, IC #3), and the left frontoparietal network (LFPN, IC #39) (Figure 1).

### Difference in FC

**Figure 2:**
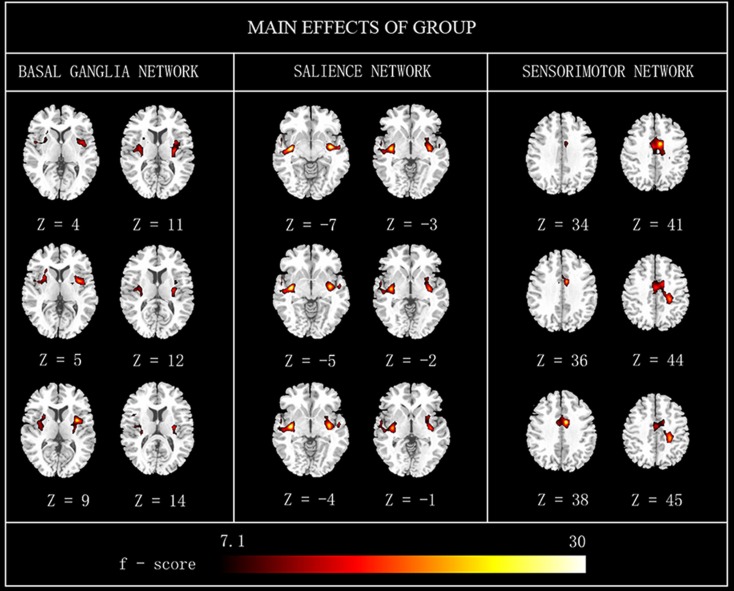
**The main effects of group on FC maps**. Most brain regions showed significant difference between groups (PD vs. HC). Hot color represents greater FC difference in the HC than in the PD. The results were obtained by two-way repeated-measures ANOVA, thresholded at *p* < 0.01 and corrected by Alphasim program. L, left; R, right; FC, functional connectivity; HC, healthy controls.

Main effects from the two-way repeated-measures ANOVA are shown in Figure 2. Brain regions showing a significant main effect of group was identified in the BGN (bilateral putamen and posterior insular cortex), SN (R putamen), and SMN [R middle cingulate cortex (MCC)] (Figure 2, PD < HC). We observed no significant main effects of state or interaction between group and state. Detailed information of regions with differences in these three networks is shown in Table 2.

**Table 2:** **Significant main effects of group between PD and HC**.

	Component	Region	Laterality	BA	*f* Score	MNI coordinates (mm)			Cluster (voxels)
						*x*	*Y*	*z*	
Main effect (group)	BGN	Putamen	L	48	7.35	−27	−5	9	210
		Insula	L	48	9.54	−33	−9	9	
		Putamen	R	48	7.78	28	−13	9	266
		Insula	R	48	21.33	39	2	7	
	SN	Putamen	R	48	21.04	29	6	−4	97
	SMN	Ciglum_Mid	R	23	−26.87	6	−4	38	233

**Figure 3:**
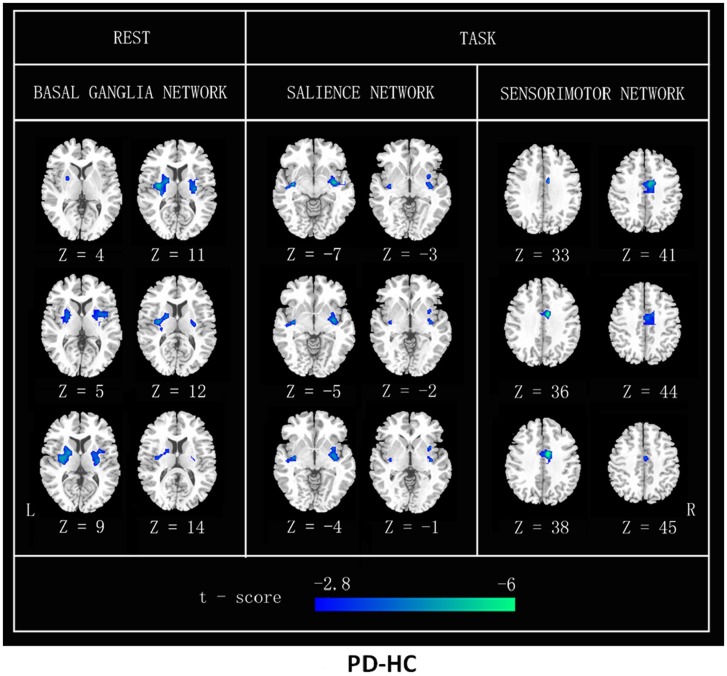
**FC differences between patients with PD and HC (PD–HC) analyzed by a *post hoc* two-sample *t* test in resting state and heat-induced pain stimuli, respectively**. Cool color represents greater FC difference in the HC than in the PD. The results were thresholded at *p* < 0.01 and corrected by Alphasim program. L, left; R, right.

Further, *post hoc* two-sample *t* tests revealed FC differences between PD and HC in different states, respectively, in Figure 3.

### Difference in FNC

**Figure 4:**
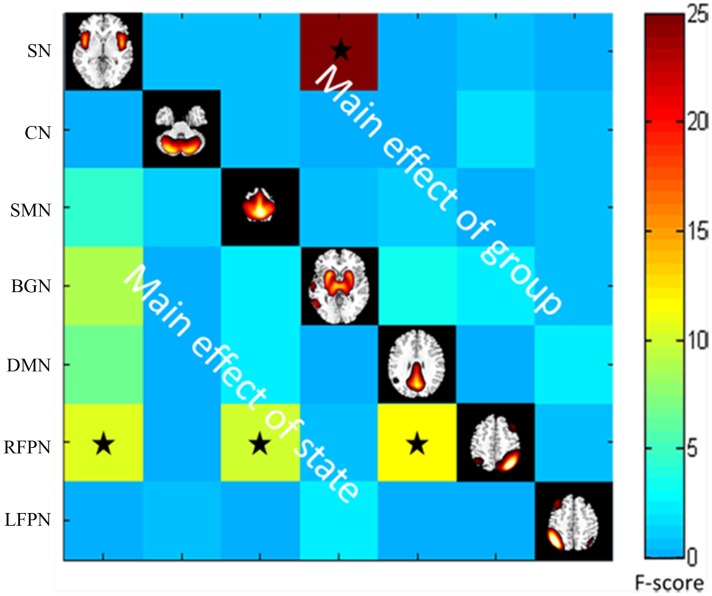
**The main effect of group and state on FNC**. The results were obtained by two-way repeated-measures ANOVA. Stars indicate pairs that survived the *t* test with an FDR-corrected *p* value threshold of 0.05.

Throughout the FNC, there was a significant difference in the main effects of group in the link between the SN and BGN (PD < HC), and in the main effects of state, including the links between the SMN and RFPN, between the SN and the RFPN and between the DMN and the RFPN (rest < task) (Figure 4).

**Figure 5:**
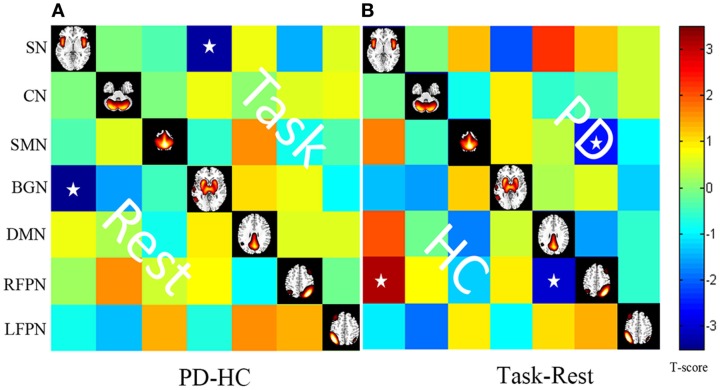
**(A)** Differences in the FNC based on *post hoc* two-sample *t* tests between patients with PD and HC (PD–HC) at task or rest state. Hot color represents greater FNC in the PD than in the HC vice-versa in cool color. **(B)** Differences in the FNC based on *post hoc* paired *t* test between task and resting states (task-rest) of patients with PD or HC. Hot color represents greater FNC in the task state than in the rest state vice-versa in cool color. Stars indicate pairs that survived the *t* test with an FDR-corrected *p* value threshold of 0.05.

*Post hoc* two-sample *t* tests (between two groups) and paired *t* tests (between two states) are shown in Figure 5.

## Discussion

By applying ICA to aggregated resting and heat pain stimuli in two groups, we successfully isolated 47 ICNs and selected 7 ICNs, including the BGN, SN, SMN, DMN, LFPN, RFPN, and the CN. These data reveal that in early drug-naïve PD patients, interconnections between the BGN and the SN are significantly reduced in either resting or task states. Furthermore, BGN intraconnections are similarly decreased in the basal ganglia (BG) region, including the bilateral putamen. Our observation also shows that in early stage PD, the FNC between the RFPN and SN, the FNC between the RFPN and DMN, and the FC in supplementary motor area (SMA) are severely disturbed during heat-induced pain stimuli.

### The characteristic BGN of PD

The BG plays a role in the pathological mechanisms underlying PD. Consistent with this view, we found a significantly decreased FNC between the BGN and the SN for both resting and task states in the PD group compared with the control group. This finding provides evidence to suggest the relatively stable FNC abnormalities associated with the BGN which are not easy to be altered by external stimuli in early drug-naïve PD patients. Smith et al. also found a close correspondence between major brain networks and their sub-networks during independent analyses of the resting and activated brain (Smith et al., 2009). The BG is involved in many neuronal pathways, including those associated with emotional, motivational, associative, and cognitive functions (Brooks and Pavese, 2011). Aside from the motor abnormality in patients with PD, emotion also influenced the FC of the putamen in PD patients with/without depression (Luo et al., 2014b). In this study, we identified the FC of the putamen nucleus in the SN, which is consistent with the idea that BG circuits modulate rapid information processing. Reduced connectivity between the SN and the BGN in the PD group may be induced by dopaminergic treatments of this complex network. This study provides evidence of a consistent disturbance in the BGN of patients with PD, suggesting that BGN connection may play an important role in PD pathogenesis.

The FC inside the BG region was also significantly decreased in the PD group compared with controls at rest or task. We found significantly reduced FC in the bilateral putamen and posterior insula regions of the BGN in the PD patients at rest and a reduced putamen in the SN of the PD patients at task. Our results are consistent with a previously study (Szewczyk-Krolikowski et al., 2014). Wu et al. (2009) also reported reduced regional homogeneity and a reduced degree of seed-based connectivity in a few voxels of the putamen in the PD group. The putamen nucleus processes information that is important for the SN (Garcia-Garcia et al., 2013), consistent with possibility that circuits in the BG modulate the rapid processing of information. Taken together, these results provide further evidence that the putamen and the posterior insula play an important role in PD.

In PD, changes in FC are most apparent in the BGN. Taken together, our findings suggest that BGN may be more functionally embroiled in early drug-naïve PD. The BGN may also be related to dysfunction in the cortico-striatal network.

### Relationship between the BGN and heat pain in PD

Basal ganglia network is also a very important portion in pain pathways, which include the lateral and medial pain pathways. The medial pain system was thought to be involved in evaluation of pain quality and integration of its affective aspects. The lateral pain system was considered to facilitate the sensory-discriminative component of pain (Wasner and Deuschl, 2012).

Substantia nigra neurons project to the prefrontal cortex, amygdala, cingulate cortex, thalamus, putamen, and the spinal cord. Together with the primary sensory cortex (S1), secondary sensory cortex, and insula, these brain structures form a network that transmits and processes pain stimuli. Nociceptive nerve fibers pass through the lateral and medial nuclei of the thalamus and project to higher cortical regions (Brooks et al., 2002). The efferent pathways link the BGN to pain-processing area. A significant decrease in the PD participant’s FNC between the BGN and the SN during the heat pain stimuli compared to that of the controls suggests that the BGN is potentially disturbed by PD and might result in the dysfunction of the pain network, as mentioned above. Furthermore, a significant decrease of FC maps in the SMN region in the patients at task also indicates that the BGN is disturbed in PD patients. The decreased region of SMN was surrounded by MCC, which was normal on the FC maps of the patients at rest but visibly reduced on those of the patients at task. Our previous study (Tan et al., 2015) identified areas involved in pain perception during heat-induced pain stimuli, including the S1 and the cingulate gyrus. The SMN is critical for sensory-discriminative processing, which is related to environmental monitoring and response selection. Cerebrocortical activity is heavily influenced by interactions with the BG (Starr et al., 2011). We inferred that abnormalities in the BG region were due to the interaction of decreased activity in the SMN. The putamen was also decreased in the SN FC of the PD patients during the heat pain stimuli compared that of the controls. The putamen is one of the major sites of cortical input into BG loops and is frequently activated during pain. This suggests that the putamen may be involved in altering brain function related to pain in PD.

Through the analysis of the relationship between the BGN and pain networks in PD patients, we inferred that the BGN plays a central role in the pain network and proved that BGN is potentially disturbed by PD.

### Function of the RFPN in PD and during heat pain

Pain, the indication of a harmful external stimulus, interrupts the resting state of the human brain (Tagliazucchi et al., 2010). The SMN, frontoparietal attentional network, the SN, and the DMN may be targets for this type of stimulus. When comparing the task and resting states in the control group, we detected increased connectivity between the SN and the RFPN and decreased connectivity between the DMN and the RFPN. The PD group did not display these connections, but we did observe decreased connectivity between the RFPN and the SMN.

Network dynamics between the DMN, frontoparietal network, and the SN are more robust in the right hemisphere (Sridharan et al., 2008; Chen et al., 2013). In humans, these three large-scale neural networks play important roles in cognitive and emotional information processing. In our previous study (Kong et al., 2013), fMRI data revealed increased activity in the frontoparietal network, including the right frontal and parietal cortices, during pain and the anticipation of pain. Furthermore, activation was stronger in the right frontal and parietal cortices than on the left side. Our data are consistent with previous observations that cognitively demanding tasks typically increase activity in the RFPN and the SN and decrease activity in the DMN (Sridharan et al., 2008).

Connectivity between the RFPN and the SMN was decreased in the PD group at task. PD disrupts the connection between the RFPN and the SMN and allows the development of abnormal connections. The frontoparietal control system mediates cognitive control and decision-making processes. Recent neuroimaging evidence suggests that the gait freezing observed in PD is due to dysfunctional interactions between frontoparietal cortical regions and subcortical structures (Shine et al., 2013). Compared with the controls, PD patients showed less activity in the SMA in this study. Several studies have observed decreased activity in the SMA (Rascol et al., 1992; Wu et al., 2009) and increased activity in the cerebellum and frontal cortex (Conte et al., 2013). These findings may reflect abnormal interactions between the frontoparietal control system and the sensory-motor system in PD.

Our study suggests that the RFPN may be involved in processing pain, which could be further investigated using task-based cognitive neuroscience methods. Moreover, if PD disrupts normal pain processing by the RFPN, it may be a strong contributor to central nervous system-mediated pain dysfunction.

With regard to the limitations of the study, ICA, as a data-driven method for fMRI analysis, was used to detect pain-related and PD-related ICNs. ICA results might be influenced by the model order estimation. Second, the small sample sizes in this study might reflect the PD abnormalities partly. Furthermore, limited sample size might also influence to detect the relation between altered connectivity in FNC and clinical characteristics (UPDRS and MMSE), which was not observed in this study. Future studies should include more samples to determine these mechanisms.

In conclusion, we analyzed the FC of intra- and inter-networks to identify abnormalities by comparing PD patients with controls during a resting state and a heat-induced pain task state. Our results reveal that the BGN plays a role in the pathological mechanisms underlying PD, and RFPN likely contributes greatly to harmonization between intrinsic brain activity and external pain stimuli.

## Conflict of Interest Statement

The authors declare that the research was conducted in the absence of any commercial or financial relationships that could be construed as a potential conflict of interest.

## Footnotes

^1^http://www.fil.ion.ucl.ac.uk/spm

^2^http://icatb.soureforge.net

^3^http://mialab.mrn.org/software/#fnc
